# Frailty Prevention Care Management Program (FPCMP) on Frailty and Health Function in Community-Dwelling Older Adults: A Quasi-Experimental Trial Protocol

**DOI:** 10.3390/healthcare11243188

**Published:** 2023-12-17

**Authors:** Chia-Hui Lin, Ming-Yi Liu, Nan-Fu Chen

**Affiliations:** 1 Department of Nursing, Chang Gung University of Science and Technology, Chiayi Campus, Chiayi City 61363, Taiwan; 2 Department of Senior Welfare and Services, Southern Taiwan University of Science and Technology, Tainan City 710301, Taiwan; liumiyi@stust.edu.tw; 3 Department of General Education, Chang Gung University of Science and Technology, Chang Gung Medical Foundation, Chiayi City 61363, Taiwan; nfchen@mail.cgust.edu.tw

**Keywords:** frailty, prevention care management, Total Resistance Exercises, senior fitness test, nutrition assessment

## Abstract

Background: Frailty often results from deteriorating muscle strength and decreased physical function in older adults. Frailty includes not only physical components, but also psychological and social aspects. Previous research has shown that exercise programs, especially resistance exercises combined with nutritional care, can reduce frailty. Objectives: This study aimed to develop a Frailty Prevention Care Management Program that prevents frailty and improves physical activity and nutrition compared to usual care for community-dwelling older adults. Methods: A quasi-experimental and single-blinded trial with a non-equivalent control group using a before-after design will be performed involving Frailty Prevention Care Management Program interventions, taking place both at the communities. Participants will be divided into two different intervention groups and two control groups. All groups will be assessed three times: at baseline, immediately after the intervention, and 3 months post intervention. A total of 72 community-dwelling older adults are recruited. This intervention includes an exercise program (design TRX program) and nutritional education. The control group will not receive any specific exercise training. The primary outcome shall comprise the effect of the Frailty Prevention Care Management Program on frailty using the Taiwanese version of the Tilburg frailty indicator. Secondary outcomes include the effect of physical activity using the Senior Fitness Test and nutrition measures using the Mini Nutritional Assessment-Short Form. A generalized estimating equation is constructed to analyze the effects of the intervention. Conclusions: This trial will provide vital information to guide interventions to improve outcomes (frailty, physical activity, and nutrition) and inform the integration of nutrition and TRX exercises in community-dwelling older adults.

## 1. Introduction

According to a systematic and meta-analysis research involving data from more than 120,000 older adults from 28 countries, the pre-frailty incidence rate per 1000 person-years for people over 60 years old was 43.4, and the frailty incidence rate was 150.6 [1]. As older adults grow older, frailty problems also appear, resulting in decreased daily activities, decreased muscle strength, a poor sense of balance, chronic diseases, and other unhealthy conditions, aggravating the pre-frailty of older adults and leading to hospitalization, falls, and death [2].

Frailty reduces the quality of life in older adults [3]. Fried et al. (2001) identified the symptoms of frailty as follows: weight loss, fatigue, low grip strength, slow gait, and low physical activity [4]. Frailty was defined as the presence of at least three of these symptoms. Frailty is usually caused by the deterioration of muscle strength and decline in physical function, often due to a lack of physical activity, chronic nutritional deficiencies, depression, or inadequate support systems [5]. The concept of frailty includes not only physical components, but also psychological and social aspects [6]. Brown et al. (1995) proposed that vulnerability is a multidimensional theoretical framework with common influencing factors. This cluster includes physical, cognitive/psychological, socioeconomic, nutritional, and social factors, as well as disease and aging, reflecting a bio-psycho-social-spiritual view of health [7]. Many researchers have indicated that frailty is multidimensional and that its definition should comprise nutrition, cognition, mentality, ADL, and other aspects. Therefore, using this protocol, we aim to adopt findings and assessment tools (Tilburg frailty indicator (TFI)) from previous studies, using the multi-dimensional perspective of frailty. In addition, some research studies have found that physical frailty and social frailty are related. A higher prevalence of muscle weakness and loss of skeletal muscle mass in participants with social frailty was shown than in those without [8]. As suggested by Cook et al. (2017), due to the multidimensional nature of frailty, the combination of physical, psychological, and social frailty is more likely to contribute to disability and mortality than physical, psychological, or social frailty alone [9]. To summarize the above, uncovering the potential pathways of frailty in combination with its three domains (physiological, psychological, and social levels) is essential and could increase our understanding of frailty from a more comprehensive perspective.

The symptoms of frailty mentioned above are also easily observed in the lives of older adults in the community. Intervention to prevent or delay frailty has important benefits for older adults. If the early stage of frailty can be detected and intervention measures can be taken in the early stage, this can delay or prevent frailty from causing adverse health effects. Intervention to prevent or delay frailty has essential benefits for older people, health services, and the current government focus on long-term care.

Much research has been conducted on nutrition, physical activity, exercise, social support, and multiple interventions to prevent frailty [10,11]. One study showed that taking a twice-daily oral supplement rich in energy, protein, fiber, vitamin D, and calcium effectively enhanced physical performance when combined with an exercise program. Exercise programs, particularly resistance exercises combined with nutritional care, can reduce frailty in primary care settings [12,13]. Another randomized controlled study on nutritional support and physical training in frail and pre-frail patients showed that combining these interventions effectively enhanced strength and physical performance [14,15].

Exercise interventions appear to be essential components of multidomain interventions. This program combines strength, resistance (aerobic), balance, and flexibility training. It has been shown to significantly improve functional capacity, which is the key to maintaining independence from tools of daily living and essential activities [16]. The characteristics of an exercise program (i.e., frequency, intensity, duration, and type of training) affect its effectiveness [13]. To better understand this issue, exercise interventions reported in the literature were analyzed using the American Institute of Physical Education’s Frequency, Intensity, Time, Type, Volume, Progression (FITT-VP) framework [17].

Based on recent research and the World Health Organization’s 2020 guidelines on physical activity and sedentary behavior, multi-component physical activity may reduce the risk of fall-related injuries in older adults, suggesting that they should include it in their weekly physical activity. They should engage in moderate- or higher intensity multi-component physical activity on 3 or more days per week to enhance functional capacity [18], 30–45 min at a time, for at least 5 months [19]. Studies have also confirmed that physical activity, especially enhancing muscular strength, balance, flexibility, and muscular endurance, is an effective treatment for frailty [20]. Daily activities and functional training are becoming increasingly important in older adults.

Total Resistance Exercises (TRX) are a core stability training program. This training mode combines physical function and strength. Body strength and balance sensitivity training can be performed [21]. Previous studies have found that TRX training can be used for trunk muscles, such as the transverse abdominis, internal oblique, rectus abdominis, multifidus, and vertical spine muscles [22,23]; it builds strong muscles by burning fat located on the muscle, strengthens the chest muscles and legs, and increases flexibility and endurance [24]. Thus, TRX training appears to provide greater benefits than traditional resistance training. Consider the following advantages of using TRX as a health function for training older adults: practicality, simplicity, ease of use, and small footprint for performing various exercises [25]. Aslani et al. (2018) found that after five weeks of training, increasing the strength of the lower body muscles and increasing the activity of the core stabilizer muscles through TRX training improved the balance performance of the subjects [26]. Exercise can effectively prevent or delay frailty and maintain physical function. Gaedtke and Morat conducted a 12-week training program on 11 old adults and concluded that strength and balance were significantly increased [21]. For older adults with total knee replacement, a six-week sling training led to significant quadriceps and hamstring strength improvements of 77% and 56%, respectively [27]. In addition, applying group-directed intervention was the choice for the delivery of our present multi-domain intervention. This is because it is known to promote social participation, physical activity, psychological factors, and social relationships [21].

However, there is still a lack of knowledge about the effects of sling training in frail older adults. Indeed, so far, there are no published studies on TRX exercise combined with a nutrition program in frailty older adults. Therefore, this study aimed to develop a Frailty Prevention Care Management Program that prevents frailty and improves physical activity and nutrition compared to usual care for community-dwelling older adults. It has three objectives:

(1) To develop and implement a program for the Frailty Prevention Care Management Program of community-dwelling older adults;
(2) To assess the impact of the intervention regarding frailty (physical, psychologic, and social domain), health function, and nutrition in the two groups (experimental and control group);
(3) To assess the impact of the Frailty Prevention Care Management Program intervention regarding frailty (physical, psychologic, and social domain) and nutrition immediately post-intervention and 3 months post intervention in the two groups.

## 2. Materials and Methods

This quasi-experimental and single-blinded trial with a non-equivalent control group uses a before-after design. The FPCMP-Old Age program trial will take the form of a non-randomized, two-group (experimental and control group) trial, with repeated measures at three time-points to improve the community effects of different levels of frailty, health function, and nutrition (Table 1). Cluster RCTs are increasingly used, particularly in primary care research [28], in which groups of participants rather than individual participants are randomized. However, practical challenges remain, such as an increased sample size and complex recruitment procedures. Due to time and resource limitations, this study did not choose to use cluster RCT at the level of communities.

When collecting research samples, the “community” was deliberately used as the unit of collection. Considering that this study recruits four units (including the experimental group and the control group), community units need to be involved. The experimental group and the control group cannot be recruited from the same unit, and this study is prone to contamination problems. Another reason why this study cannot use random assignment is to consider the four community units that received the case. If random assignment is used, there is a chance that strongholds with better or worse functionality will be assigned to the same group (experimental group or control group). Therefore, this study adopts a match to make the health attributes of the two groups of participants close. It was expected that the proportion of control confounding factor allocation will be balanced within the two groups. Therefore, to control or reduce the impact of confounding factors on research, the community was selected, and the experimental and control groups will be recruited from four different communities, but with similar health attributes (Chiayi and Yunlin County).

### 2.1. Participants and Eligibility

The scope of this study was to accept cases in Chiayi and Yunlin County. Four convenient samples, two experimental groups, and two control groups of people meeting the inclusion criteria will be drawn from four communities, respectively. These four communities are similar in the main aspects, such as population density, economics, and level of community health services. With the support of the local health bureau, the program was accepted by the communities. The research nurses independently completed the data collection, and data collection was completed through one-on-one interviews.

The participants could not be masked after allocation because of the nature of the intervention. The research nurses were similarly not masked because they had to allocate patients to the experimental group and monitor the integrity of the intervention. This study blinded the athletic trainers and did not know that which community participants were the experimental group, because athletic trainers had no role in recruitment, outcome assessment, or data analysis for this study.

#### 2.1.1. Establish a Cooperative Relationship with the Local Township Head, Village Head, or Person in Charge of the Base

We invited the township head, section chief of the township office, village head, or person in charge of the community to discuss the purpose of this research. The communities were positive and pleased to collaborate with our program. Further, we explained the content of the activities, the participants, and procedures of accepting cases, among others, to obtain the support of relevant units in the community and establish a cooperative relationship.

#### 2.1.2. Preliminary Solicitation of Patients Willing to Participate in the Testing

**Figure 1:**
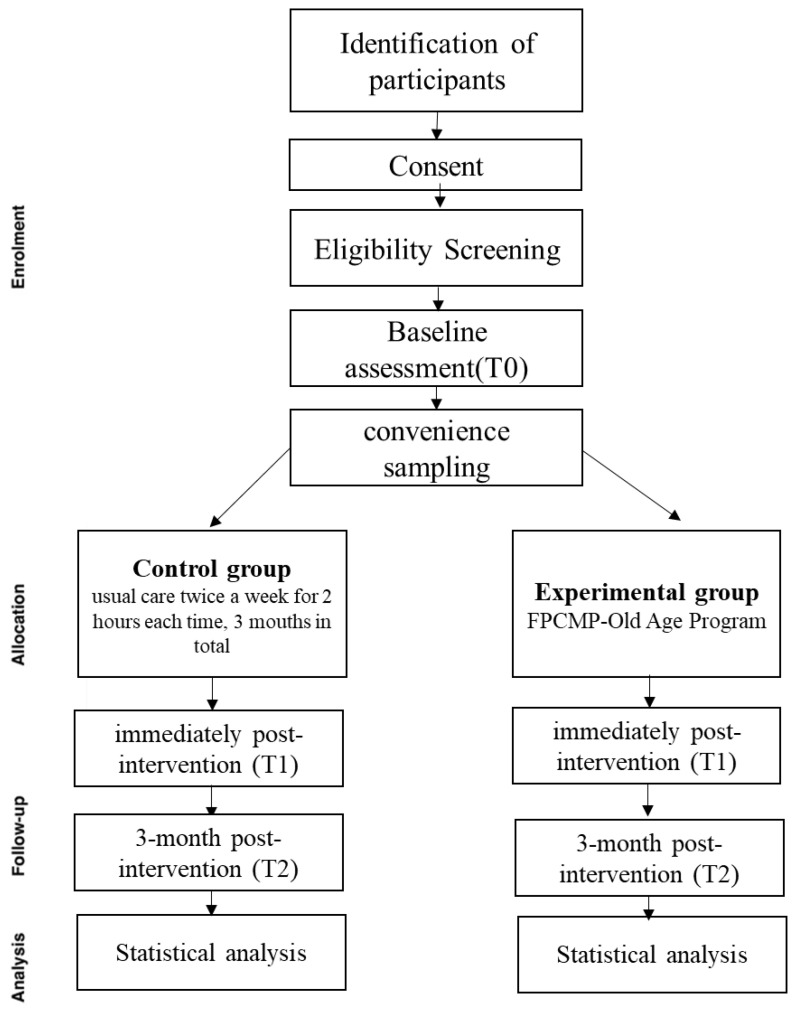
Overview of the flow of participants through the FPCMP-Old Age Trial. Adapted from the CONSORT diagram.

After the invitation process, the information sheets will be distributed and explained. Those individuals who agree to participate will be requested to provide written informed consent, and subsequently, they will be further screened to identify their eligibility to participate. Through questionnaires, case capital data, frailty status, physical function, nutrition, and other tools for older adults in the community were collected. Primary and secondary study outcomes will be collected for the intervention and control groups at baseline assessment (T0), the week immediately post intervention (T1), and at 3 months post intervention (T2; See Figure 1).

The study protocol was approved by the regional Chang Gung Medical Foundation 202202269B0 (See Supplementary File S1). The study protocolwas registered in the ClinicalTrial.gov-register (NCT05883423).

### 2.2. Recruitment of Participants

The study adopted convenience sampling and selected community-dwelling older adults willing to participate in the study in Chiayi County and Yunlin County in Taiwan.

Inclusion criteria are (a) age 60 years or older, (b) ability to speak and understand Chinese, and (c) voluntary participation in the activities of this course. Exclusion criteria were (a) severe chronic diseases (such as heart disease, end-stage kidney disease, cancer) or severe illness, among others, which mean that older adults cannot exercise; (b) severe visual and hearing impairments or using assistive devices without communication barriers; (c) severe bone and joint diseases (e.g., severe osteoporosis, severe knee, or shoulder joint degeneration); (d) arrhythmia requiring drug treatment or a cardiac pacemaker; and (e) severe cognitive impairment (clinical dementia rating ≧ 1) [29].

## 3. Outcome Measures and Measurement Procedures

The primary and secondary outcomes will be assessed at baseline, immediately post intervention, and 3 months post intervention (Table 1).

### 3.1. Description of Outcome Measures

#### 3.1.1. Baseline Measures

Baseline data will include age, sex, education, marital status, residency status, and the experience of one or more events. Most of these data will be assessed through face-to-face interviews with patients in their homes. Data routinely documented by the respective home care services were collected during visits to the home care services.

##### Chronic Disease and Health Problems

The research tools refer to the ‘Chronic Disease and Health Problems Scale (CDHPS) edited by Pao-Hsia Pan (2012) [30]. There are 21 chronic diseases and health problems identified. Respondents were asked to self-evaluate their chronic diseases and the number of symptoms of health problems and to explore the severity of chronic diseases and health problems that interfere with daily life. The scoring method for the severity of interference was 0 points for ‘no inconvenience’, 1 point for ‘slight inconvenience’, and 2 points for ‘slight inconvenience’. The total score ranged from 0 to 42 points.

#### 3.1.2. Primary Outcome

##### Frailty

Frailty was assessed using the Taiwanese Tilburg frailty indicator (TFI-T) [31]. It is divided into three parts with a total of 15 items. The first part measures physical frailty (score range 0–8 points), including unexpected weight loss, physical fitness, difficulty in walking, balance, visual problems, hearing problems, hand strength, and physical fatigue. The second part assesses mental frailty (score range 0–4 points), including cognition, depressive symptoms, anxiety, and coping abilities. The third part is social-level frailty (score range 0–3 points), including living alone, social relationships, and social support, with 0–1 point for each question and a total score of 15 points. The higher the score, the more severe the frailty. The cut-off score of the frailty problem measured using the TFI-T for older adults in the Taiwanese community is 5.5 points, and a score above 5.5 is defined as frailty. The tool has good reliability and validity [31].

#### 3.1.3. Secondary Outcomes

##### Health Function (Physical Function—Senior Fitness Test, SFT)

Physical function concerns the ability of subjects to perform daily activities independently without excessive fatigue and safety concerns (such as climbing stairs easily, walking, bathing, changing clothes, taking care of meals, shopping, and the flexible use of wheelchairs), and the aim was to detect the muscles of the elderly strength, muscular endurance, cardiorespiratory endurance, body flexibility, balance, coordination, and reaction time. The Senior Fitness Test was used to assess the motor skills of older adults [32]. This test assessed strength, flexibility, balance, and endurance [33,34]. It includes the following tests: chair sit-and-reach test, back scratch test, chair stand test, The Biceps Curl Test, 8-foot up-and-go test, and 2 min step test [35]. Currently, the STF tool is widely used in many countries to assess the physical function of older adults. It has been translated into Chinese and other languages and verified for reliability and validity. It can be used by older adults in Chinese culture [34].

##### Health Function (Nutrition—Mini Nutritional Assessment-Short Form, MNA-SF)

The Mini Nutritional Assessment-Short Form has been validated as a screening tool with high sensitivity (97%) and specificity compared with the MNA complete test. The MNA-SF contains only 6 of the original 18 items on the MNA and takes approximately five minutes to execute. The questions concern body mass index, recent weight loss, appetite or eating problems, mobility impairment, acute illness or psychological stress, and dementia or depression. Each question is scored on a scale of 0 to 2 or 3 for an overall MNA-SF score of 14. Patients with a score of 12–14 have normal nutritional status, and those with a score of ≤11 are at nutritional risk or malnutrition [36,37].

## 4. Therapeutic Intervention

### 4.1. Study Protocol

This protocol employs relevant standard protocol items for clinical trials according to the SPRTI 2013 statement (Supplementary File S2).

### 4.2. Experimental Group

**Figure 2:**
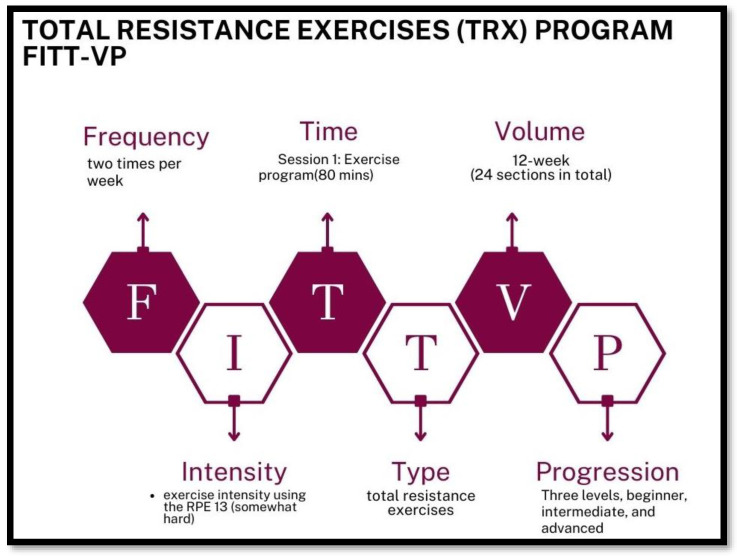
Total Resistance Exercises (TRX) program with FITT-VP.

**Figure 3:**
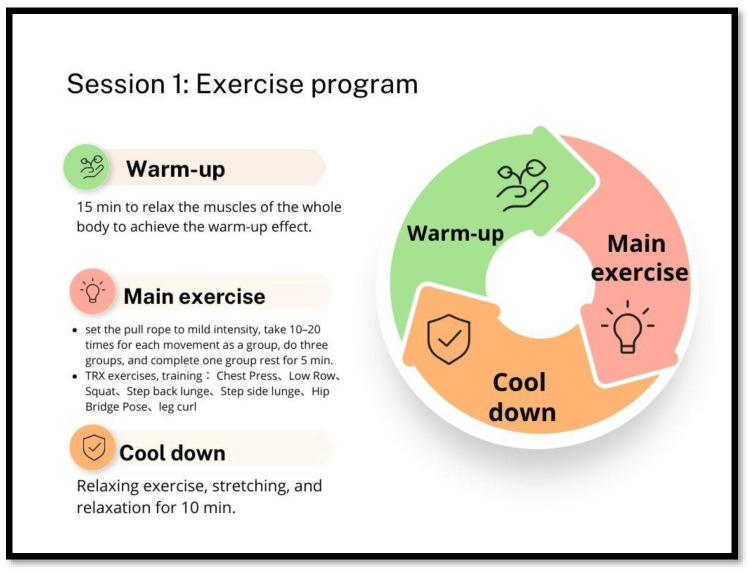
Total Resistance Exercises (TRX) program protocol.

Participants in the experimental group will receive a Frailty Prevention Care Management Program (FPCMP-Old Age Program) for 12 weeks. This intervention included an exercise program (design TRX program) and nutritional education. The main elements of the FPCMP-Old Age Program intervention are outlined below in Figure 2 and Figure 3.

#### 4.2.1. Frailty Prevention Care Management Program-Old Age Program

The Frailty Prevention Care Management Program aligns with promoting frailty prevention among older adults in the community. Experts in nursing, long-term care, sports, and functional fields and scholars designed the FPCMP-Old Age Program. The program mainly refers to Ha and Park (2020) and extends the 12-week FPCMP-Old Age Program development, with 120 min per session two times per week [12] (See Supplementary File S3), scheduled from July 2023 to the end of September 2023.

#### 4.2.2. Session 1: Exercise Program-Total Resistance Exercises (TRX) Program

The main purpose of the Total Resistance Exercises (TRX) program was to improve upper- and lower-limb muscle strength and endurance, body flexibility, and a sense of balance and coordination. The exercise program was carried out with a suspension training system, which can improve core muscle strength and physical fitness, activate more core and stabilizing muscle groups, increase joint stability, and strengthen ligaments [38]. It was suitable for use by groups of all ages [39]. The implementation of TRX was not limited by insufficient resources such as venue space, equipment settings, and teachers, because it can be used in almost all environments, and it was convenient and accessible. The TRX was characterized by two straps hanging from an anchor point. To ensure safety throughout training, it was essential to have an anchor point that supports the body weight [21].

The exercise program of this research was designed in terms of frequency; the group exercise training will be implemented twice a week. In terms of intensity, participants will be reminded to consciously feel their body so that they can feel ‘a little sore, a little tight, but not too tired or too tight. The exercise intensity uses Borg’s Rating of Perceived Exertion (RPE) [39]. Participants in the Total Resistance Exercises (TRX) program will aim to achieve a target RPE of 13 (somewhat hard) while exercising.

All movements must maintain a natural upward extension of the spine, slightly retract the jaw, relax the joints, and keep them natural—the principle of breathing without holding one’s breath. Before implementing the exercise, it was necessary to perform a step-by-step inspection, test the venue equipment, and demonstrate and explain the essentials of the action.

##### Each Course Is Divided into Three Parts

(1) Warm-up: A total of 15 min to relax the muscles of the whole body to achieve the warm-up effect.
(2) Main exercise: According to the physical function of older adults, they will start training from the simplest movements and angles, set the pull rope to mild intensity, take 10–20 times for each movement as a group, perform three sets as a group, and complete one group rest for 5 min. The following was a description of the scheduled execution movements: performing upper limb TRX exercises, such as ‘Chest Press’ movements (training pectoralis major, anterior deltoid muscles, and triceps brachii) and ‘Low Row’ movements (training back muscles, rear deltoid, and biceps); performing lower body TRX exercises, as as ‘Squat’ (lower body muscles) and ‘Step Back Lunge’ and ‘Step Side Lunge’ (lower body muscles, stability, balance, single leg stability, and power hip adductors). Further, they will perform the ‘Hip Bridge Pose’ (triceps, core, hip flexors, quadriceps, and hamstrings), ‘Leg Curl’ (biceps and core muscles), and eight other movements [40].

The program was divided into three levels, beginner (5–10 times for each group during the first five weeks), intermediate (10–15 times for group, from the 6th week to the 10th week), and advanced (15–20 times for each group, from the 11th week to the 12th week). However, all participants in this study had comparable ability at baseline; therefore, all participants were started at the beginner level and then moved to intermediate and advanced levels as a group. During the exercise, the number of exercises will be gradually increased according to the participant’s physical condition to improve the participant’s muscular endurance.

(3) Cool down: Relaxing exercise, stretching, and relaxation for 10 min.

#### 4.2.3. Session 2: Nutrition Program

The nutritional intervention course included individual nutritional assessment (3 times), education (14 times), and counseling (8 times) for 40 min each time. The main method was to educate the elderly to increase their intake of calories, proteins, calcium, and vitamins [41], considering the age, sex, height of the participants, body weight, and physical activity to assess caloric needs. Participants received a healthy lunch set with a vegetable and protein plate with four compartments, a bowl for rice and fruit, a glass for milk and juice, and a tablespoon [42]. A colored placemat was used to indicate the amount of individualized food on the tableware, helping participants know that they should consume sufficient rice, protein, fruits, vegetables, milk, and nuts daily [42].

### 4.3. Control Group

Participants in the control group will not receive any specific exercise training (i.e., maintain their usual physical activity).

## 5. Statistical Analysis

### 5.1. Data Analysis

Data processing and statistical analyses were performed using the IBM SPSS software (version 23.0; SPSS, Inc., Chicago, IL, USA) for Windows. The mean and standard deviation were used for continuous data. The number, percentage, and homogeneity of categorical data were tested using the *t*-test, and the chi-square test was used for categorical data. The generalized estimating equation (GEE) was used to analyze the two groups before and after, immediately post intervention, and 3 months post intervention [43]. The consideration for choosing the GEE is to control some possible interference factors, deal with multiple repeated measurements of the same case, and explore the primary interaction among variables [44]. We used simple imputation for missing data, including the last or baseline observation carried forward as close to real time as possible during the study [45].

### 5.2. Sample Size

The sample size for each arm was calculated using the statistical power of G Power [46]. The parameters applied included (a) an alpha level of 0.05 and (b) a statistical power of >80%. The effect size (ES) is estimated based on previous studies [47]. At least 30 subjects in each group were sufficient. Considering a 20% dropout rate, 36 participants in each group were recruited for the trial, therefore there were 72 participants at baseline.

### 5.3. Data Management

To truly protect the identity of the research participants and prevent a chain reaction caused by relevant personal information, this study will anonymize all the collected information and promise to keep it confidential. All information that may identify the participants will be used either anonymized or using a codename replacement. The questionnaire data will also be properly kept. Data will be destroyed 5 years after publication.

## 6. Discussion

This quasi-experimental study examined the effects of the FPCMP-Old Age Program intervention on frailty, physical activity, and nutrition in two groups of older adults (experimental and control). In the current study, many exercise programs were used in the community, including Vivifrail [16], Tai Chi [48], resistance exercises [49], and TRX [21]. These interventions based on exercise interventions should be highly recommended in treatment and prevention to improve physical function, functional capacity, and quality of life in frail older adults.

Many TRX studies had been conducted in adolescent athletes for performance enhancement [24,50], weight management [51], and as a cardiovascular and resistance training exercise [52]. The above-mentioned studies have shown that TRX exercises can train and improve strength, endurance, coordination, flexibility, and explosive power; reduce body fat, systolic and diastolic blood pressure, and waist circumference; and increase muscle mass [50,51,52]. However, some researchers reported that TRX training had no effect on cardiorespiratory endurance capacity [50].

Some of these studies developed short-term practical applications and small samples. Few experimental studies have been conducted on using TRX exercises to prevent frailty in older adults, and empirical results are lacking [21,26,50,51,52]. Considering whether the older adults have enough physical strength to perform TRX exercises safely, this study attempts to design a multiple-intervention program to integrate TRX exercises and nutritional intervention into the FPCMP-Old Age Program. We expect to develop interventions appropriate for the community domain to benefit long-term care and sustainable community development.

An additional advantage of this community-based intervention is that it will be conducted in a natural setting, allowing respondents to integrate changes into their daily lives in a real-life context. The intervention being examined is readily transferable to several professional groups in health care such as nurses, dietitians, and physiotherapists.

This study has several limitations. First, quasi-experimental studies’ lack of randomization may lead to selection bias. The main consideration is that communities willing to participate must obtain the consent of all older adults to participate in the program. In addition, the random sampling method is likely to cause dissatisfaction among the older adults in the community, because most older adults hope to participate in this project together. The sample was not randomized for pragmatic and ethical reasons, as it might be considered unethical not to prescribe exercise to frail older adults [53]. However, the lack of randomization resulted in the results being contaminated with confounding variables. Second, a potential limitation of this study is that participants and research nurses could not be blinded, as it would be impossible to blind them in non-drug trials. Therefore, performance bias may be unavoidable. In addition, to obtain the consent of all participants in the experimental group, participants must be informed of the risks of participating in the study. In this case, the “Hawthorne effect” may occur in the experimental group.

### FPCMP-Old Age Program Development Challenges and Solutions

Older adults should exercise cautiously, as their age and potential health conditions, such as heart disease, high or low blood pressure, diabetes, and severe degeneration of bones and joints, require careful consideration, regardless of their physical health status [54]. Professional medical personnel first evaluated age, past medical history, the medicine used, and whether there were risk factors for coronary artery disease; they also asked whether the older adults had experienced discomfort symptoms and restrictions during exercise, and measured blood pressure, heart rate, and blood oxygen before the activity exercise [54]. Implementing the FPCMP-Old Age Program should avoid injuries in participants, according to Gaedtke and Morat (2015), who studied the application of TRX [21]. It is worth noting that older adults are prone to knee, waist, or hip injuries owing to incorrect posture [21]. According to the recommendations, before each training session, the coach checked safety points to avoid injuries. The training time for older adults should be 30–45 min to ensure adequate training and physical recovery [55]. The ratio of the number of older individuals guided by trained coaches was 1:5 [21].

However, this intervention plan will face some practical challenges and limitations when planning to promote TRX exercises to the community, such as athletic trainers needing to receive TRX exercise training, health indicators of older adults (e.g., severe osteoporosis must be excluded), establishing safety standards, and a safe environment created (use floor mats or knee pads) to ensure the safety of the participants.

## 7. Expected Results and Possible Benefits

- Exercise and adequate nutritional support appear to have promising effects on preventing frailty and improving physical activity and overall functioning, with significant effects on physical function and preventing frailty in older adults.
- Provide an exercise prescription-FPCMP-Old Age Program to prevent frailty in older adults and verify its effectiveness through the intervention of this study.

## 8. Conclusions

This trial will provide vital information to guide interventions to improve outcomes (frailty, physical activity, and nutrition) and inform the integration of nutrition and TRX exercises in community-dwelling older adults. Based on the evidence, we are convinced that this intervention program will be able to prevent frailty and the adverse effects associated with it in older adults.

## Figures and Tables

**Table 1:** Primary and secondary outcomes for the participants of the study.

Outcome/Variable	Scale	Baseline (T0)	Immediately Post Intervention (T1)	3 Months Post Intervention (T2)
Descriptive variables	Demographic variables and Chronic Disease and Health Problems Scale	X		
Frailty	Tilburg frailty indicator (TFI-T)	X	X	X
Health function (1) Physical function	Senior Fitness Test (SFT)			
	Chair sit-and-reach test	X	X	X
	Back scratch test	X	X	X
	Chair stand test	X	X	X
	The Biceps Curl Test	X	X	X
	8-foot up-and-go test	X	X	X
	2 min step test	X	X	X
(2) Nutrition	Mini Nutritional Assessment-Short Form (MNA-SF)	X	X	X

X check on visit.

## Supplementary Materials

The following supporting information can be downloaded at: https://www.mdpi.com/article/10.3390/healthcare11243188/s1. Supplementary File S1. Chang Gung Medical Foundation Institutional Review Board; Supplementary File S2. SPIRIT 2013 Checklist; Supplementary File S3. Frailty Prevention Care Management Program.

## References

[1] Ofori-Asenso R., Chin K.L., Mazidi M., Zomer E., Ilomaki J., Zullo A.R., Gasevic D., Ademi Z., Korhonen M.J., LoGiudice D. (2019). Global incidence of frailty and prefrailty among community-dwelling older adults: A systematic review and meta-analysis. JAMA Netw. Open.

[2] Cesari M., Vellas B., Hsu F.-C., Newman A.B., Doss H., King A.C., Manini T.M., Church T., Gill T.M., Miller M.E. (2015). A physical activity intervention to treat the frailty syndrome in older persons—Results from the LIFE-P study. J. Gerontol. Ser. A Biomed. Sci. Med. Sci..

[3] Masel M.C., Ostir G.V., Ottenbacher K.J. (2010). Frailty, mortality, and health-related quality of life in older Mexican Americans. J. Am. Geriatr. Soc..

[4] Fried L.P., Tangen C.M., Walston J., Newman A.B., Hirsch C., Gottdiener J., Seeman T., Tracy R., Kop W.J., Burke G. (2001). Frailty in older adults: Evidence for a phenotype. J. Gerontol. Ser. A Biol. Sci. Med. Sci..

[5] Levers M.J., Estabrooks C.A., Ross Kerr J.C. (2006). Factors contributing to frailty: Literature review. J. Adv. Nurs..

[6] Gobbens R.J., van Assen M.A., Luijkx K.G., Wijnen-Sponselee M.T., Schols J.M. (2010). Determinants of frailty. J. Am. Med. Dir. Assoc..

[7] Brown I., Renwick R., Raphael D. (1995). Frailty: Constructing a common meaning, definition, and conceptual framework. Int. J. Rehabil. Res..

[8] Makizako H., Kubozono T., Kiyama R., Takenaka T., Kuwahata S., Tabira T., Kanoya T., Horinouchi K., Shimada H., Ohishi M. (2019). Associations of social frailty with loss of muscle mass and muscle weakness among community-dwelling older adults. Geriatr. Gerontol. Int..

[9] Cook M.J., Oldroyd A., Pye S.R., Ward K.A., Gielen E., Ravindrarajah R., Adams J.E., Lee D.M., Bartfai G., Boonen S. (2017). Frailty and bone health in European men. Age Ageing.

[10] Pek K., Chew J., Lim J.P., Yew S., Tan C.N., Yeo A., Ding Y.Y., Lim W.S. (2020). Social frailty is independently associated with mood, nutrition, physical performance, and physical activity: Insights from a theory-guided approach. Int. J. Environ. Res. Public Health.

[11] Quach L.T., Primack J., Bozzay M., Madrigal C., Erqou S., Rudolph J.L. (2021). The intersection of physical and social frailty in older adults. Rhode Isl. Med. J. (2013).

[12] Ha J., Park Y.-H. (2020). Effects of a person-centered nursing intervention for frailty among prefrail community-dwelling older adults. Int. J. Environ. Res. Public Health.

[13] Drennan V., Walters K., Avgerinou C., Gardner B., Goodman C., Frost R., Kharicha K., Iliffe S., Manthorpe J. (2018). Moving upstream in health promoting policies for older people with early frailty in England? A policy analysis. J. Health Serv. Res. Policy.

[14] Abizanda P., López M.D., García V.P., de Dios Estrella J., da Silva González Á., Vilardell N.B., Torres K.A. (2015). Effects of an oral nutritional supplementation plus physical exercise intervention on the physical function, nutritional status, and quality of life in frail institutionalized older adults: The ACTIVNES study. J. Am. Med. Dir. Assoc..

[15] Haider S., Dorner T.E., Luger E., Kapan A., Titze S., Lackinger C., Schindler K.E. (2017). Impact of a home-based physical and nutritional intervention program conducted by lay-volunteers on handgrip strength in prefrail and frail older adults: A randomized control trial. PLoS ONE.

[16] Casas-Herrero A., Anton-Rodrigo I., Zambom-Ferraresi F., Sáez de Asteasu M.L., Martinez-Velilla N., Elexpuru-Estomba J., Marin-Epelde I., Ramon-Espinoza F., Petidier-Torregrosa R., Sanchez-Sanchez J.L. (2019). Effect of a multicomponent exercise programme (VIVIFRAIL) on functional capacity in frail community elders with cognitive decline: Study protocol for a randomized multicentre control trial. Trials.

[17] American College of Sports Medicine (2013). ACSM’s Guidelines for Exercise Testing and Prescription.

[18] Bull F.C., Al-Ansari S.S., Biddle S., Borodulin K., Buman M.P., Cardon G., Carty C., Chaput J.-P., Chastin S., Chou R. (2020). World Health Organization 2020 guidelines on physical activity and sedentary behaviour. Br. J. Sports Med..

[19] Nash K.C. (2012). The effects of exercise on strength and physical performance in frail older people: A systematic review. Rev. Clin. Gerontol..

[20] de Labra C., Guimaraes-Pinheiro C., Maseda A., Lorenzo T., Millán-Calenti J.C. (2015). Effects of physical exercise interventions in frail older adults: A systematic review of randomized controlled trials. BMC Geriatr..

[21] Gaedtke A., Morat T. (2015). TRX suspension training: A new functional training approach for older adults–development, training control and feasibility. Int. J. Exerc. Sci..

[22] Kang H., Jung J., Yu J. (2012). Comparison of trunk muscle activity during bridging exercises using a sling in patients with low back pain. J. Sports Sci. Med..

[23] Kim J.H., Kim Y.E., Bae S.H., Kim K.Y. (2013). The effect of the neurac sling exercise on postural balance adjustment and muscular response patterns in chronic low back pain patients. J. Phys. Ther. Sci..

[24] Essam-Eldeen R.M. (2017). Influence of sling exercises (TRX) on certain physical variables and performance level of high jump for female college students. Ovidius Univ. Ann. Ser. Phys. Educ. Sport/Sci. Mov. Health.

[25] Mohamed T.S. (2016). Effect of Trx Suspension Training as a Prevention Program to Avoid the Shoulder Pain for Swimmers. Ovidius Univ. Ann. Ser. Phys. Educ. Sport/Sci. Mov. Health.

[26] Aslani M., Minoonejad H., Rajabi R. (2018). Comparing the effect of trx exercise and hoping on balance in male university student athletes. Phys. Treat.-Specif. Phys. Ther. J..

[27] Bae C.-H., Jung Y.-W., Lee D.-W., Cho S.-H. (2014). The effect of sling exercise on muscular strength and range of motion in female patients who received total knee replacement. 한국산학기술학회 논문지.

[28] Elley C.R., Kerse N., Chondros P. (2004). Randomised trials-cluster versus individual randomisation: Primary Care Alliance for Clinical Trials (PACT) network. Aust. Fam. Physician.

[29] Kelaiditi E., Cesari M., Canevelli M., Abellan van Kan G., Ousset P.-J., Gillette-Guyonnet S., Ritz P., Duveau F., Soto M., Provencher V. (2013). Cognitive frailty: Rational and definition from an (IANA/IAGG) international consensus group. J. Nutr. Health Aging.

[30] Pao H.-P. (2011). The Influence of Emotional Health Status by Healthy Dietary and Exercise Behaviors among Family Medicine Outpatient of Middle-Aged Patients.

[31] Lin C.-H., Liu C.-Y., Rong J.-R. (2021). Psychometric properties of the Taiwanese version of the Tilburg frailty indicator for community-dwelling older adults. Healthcare.

[32] Bhattacharya P.K., Deka K., Roy A. (2016). Assessment of inter-rater variability of the Senior Fitness Test in the geriatric population: A community based study. Int. J. Biomed. Adv. Res..

[33] Różańska-Kirschke A., Kocur P., Wilk M., Dylewicz P. (2006). The Fullerton Fitness Test as an index of fitness in the elderly. Med. Rehabil..

[34] Rikli R.E., Jones C.J. (2013). Development and validation of criterion-referenced clinically relevant fitness standards for maintaining physical independence in later years. Gerontologist.

[35] Liu J.-D., Quach B., Chung P.-K. (2019). Further understanding of the Senior Fitness Test: Evidence from community-dwelling high function older adults in Hong Kong. Arch. Gerontol. Geriatr..

[36] Zhang X., Zhang X., Zhu Y., Tao J., Zhang Z., Zhang Y., Wang Y., Ke Y., Ren C., Xu J. (2020). Predictive value of nutritional risk screening 2002 and mini nutritional assessment short form in mortality in Chinese hospitalized geriatric patients. Clin. Interv. Aging.

[37] Wu M.-L., Courtney M.D., Shortridge-Baggett L.M., Finlayson K., Isenring E.A. (2012). Validity of the malnutrition screening tool for older adults at high risk of hospital readmission. J. Gerontol. Nurs..

[38] Gulmez I. (2017). Effects of angle variations in suspension push-up exercise. J. Strength Cond. Res..

[39] Williams N. (2017). The Borg rating of perceived exertion (RPE) scale. Occup. Med..

[40] Dawes J. (2017). Complete Guide to TRX Suspension Training.

[41] Dent E., Lien C., Lim W.S., Wong W.C., Wong C.H., Ng T.P., Woo J., Dong B., de la Vega S., Poi P.J.H. (2017). The Asia-Pacific clinical practice guidelines for the management of frailty. J. Am. Med. Dir. Assoc..

[42] Wu S.-Y., Hsu L.-L., Hsu C.-C., Hsieh T.-J., Su S.-C., Peng Y.-W., Guo T.-M., Kang Y.-W., Pan W.-H. (2018). Dietary education with customised dishware and food supplements can reduce frailty and improve mental well-being in elderly people: A single-blind randomized controlled study. Asia Pac. J. Clin. Nutr..

[43] Liang K.-Y., Zeger S.L. (1993). Regression analysis for correlated data. Annu. Rev. Public Health.

[44] Stillman S. (2003). Review of generalized estimating equations by Hardin and Hilbe. Stata J..

[45] Bell M.L., Fiero M., Horton N.J., Hsu C.-H. (2014). Handling missing data in RCTs; a review of the top medical journals. BMC Med. Res. Methodol..

[46] Faul F., Erdfelder E., Lang A.-G., Buchner A. (2007). G* Power 3: A flexible statistical power analysis program for the social, behavioral, and biomedical sciences. Behav. Res. Methods.

[47] Zhang Y., Wu J., Wang X., Zheng G. (2022). Baduanjin exercise for balance function in community-dwelling older adults with cognitive frailty: A randomized controlled trial protocol. BMC Complement. Med. Ther..

[48] Kasim N.F., van Zanten J.V., Aldred S. (2020). Tai Chi is an effective form of exercise to reduce markers of frailty in older age. Exp. Gerontol..

[49] Fiatarone M.A., O’Neill E.F., Ryan N.D., Clements K.M., Solares G.R., Nelson M.E., Roberts S.B., Kehayias J.J., Lipsitz L.A., Evans W.J. (1994). Exercise training and nutritional supplementation for physical frailty in very elderly people. N. Engl. J. Med..

[50] Fayazmilani R., Abbasi A., Hovanloo F., Rostami S. (2022). The effect of TRX and bodyweight training on physical fitness and body composition in prepubescent soccer athletes. Sport Sci. Health.

[51] Dolati M., Ghazalian F., Abednatanzi H. (2017). The effect of a period of TRX training on lipid profile and body composition in overweight women. Int. J. Sport. Sci..

[52] Smith L.E., Snow J., Fargo J.S., Buchanan C.A., Dalleck L.C. (2016). The acute and chronic health benefits of TRX Suspension Training^®^ in healthy adults. Int. J. Res. Exerc. Physiol..

[53] Izquierdo M., Rodriguez-Mañas L., Casas-Herrero A., Martinez-Velilla N., Cadore E.L., Sinclair A.J. (2016). Is it ethical not to prescribe physical activity for the elderly frail?. J. Am. Med. Dir. Assoc..

[54] Fleg J.L. (2012). Aerobic exercise in the elderly: A key to successful aging. Discov. Med..

[55] McDermott A.Y., Mernitz H. (2006). Exercise and older patients: Prescribing guidelines. Am. Fam. Physician.

