# Supplementary material for: Frailty Prevention Care Management Program (FPCMP) on Frailty and Health Function in Community-Dwelling Older Adults: A Quasi-Experimental Trial Protocol
Source: Healthcare (Basel). 2023 Dec 17;11(24):3188. doi: 10.3390/healthcare11243188 (PMC10743154; doi:10.3390/healthcare11243188)
Supplement: Supplementary file 1 [file healthcare-11-03188-s001.zip › Supplementary File S1.pdf]

Chang Gung Medical Foundation

Institutional Review Board

199, TUNG HWA NORTH ROAD,
TAIPEI, TAIWAN, 10507
REPUBLIC OF CHINA
Tel: (03) 3196200
Fax: (03) 3494549

Date 2023/03/13

Protocol Title: Effects of application of preventive care management program on frailty and health function of community elders

IRB No.: 202202269B0

Chief Investigator(s): Lin

Executing Institution: CGUST Chiayi Campus

Duration of Approval: From 2023/6/1 To 2023/12/31

Version/Date of documents:

(1)Protocol: 2023/02/16 Version2

(2)Chinese Synopsis: 2023/02/24 Version 3

(3)English Synopsis: 2022/12/17 Version1

(4)Informed Consent Form: 2023/02/25 Version 4

(5)Questionnaire: 2023/02/16 Version 2

(6)Scale: 2023/02/16 Version2

(7)Others: [慢性身體疾病與健康問題量表授權同意書]

The IRB reviewed and determined that it is expedited review according to Study properties or behaviors in individuals or the group without causing potential discrimination to individuals or the group.

Date of Approval: 2023/03/13

Frequency of Continuing Report: Once a year

※The research will expire on 2023/12/31. Please submit the final report to the IRB for review within three months after the expiration of the trial research. For extension of the trial period, please submit the amendment and a continuing report two months before the expiration.

※The research is a community study. Please provide the participating community with research results after the completion of the research.※

The IRB is organized and operates in accordance with Good Clinical Practice and the applicable laws and regulations.

Tsang-Tang Hsieh,MD
Chairman
Institutional Review Board
Chang Gung Medical Foundation

CHANG GUNG MEDICAL FOUNDATION

199 TUNG HWA NORTH ROAD

TAIPEI, TAIWAN, 10507

REPUBLIC OF CHINA

TEL(03)3196200

FAX(03)3494549

Institutional Review Board B

2022 July - 2024 June

MEMBER	GENDER	PRIMARY SPECIALTY	AFFILIATION OF INSTITUTION
主席 ： 謝燦堂<br>HSIEH, TSANG-TANG	Male<br>男性	Obstetrics/Gynecology<br>婦產科專科	CGMF (Taipei)<br>台北長庚醫院
副主席 ： 賴瓊慧<br>LAI, CHYONG-HUEY	Female<br>女性	Obstetrics/Gynecology<br>婦產科專科	CGMF (Lin-Kou)<br>林口長庚醫院
醫療執秘 ： 林永昌<br>LIN, YUNG-CHANG	Male<br>男性	Hematology/Oncology<br>腫瘤科專科	CGMF (Lin-Kou)<br>林口長庚醫院
鄭欽明<br>CHENG, CHIN-MING	Male<br>男性	Business Management<br>企業管理	Children's Hearing Foundation<br>財團法人雅文兒童聽語文教基金會
王悅賢<br>Wang, Yueh-Hsien	Male<br>男性	Law<br>法律	Baker & McKenzie, Taipei<br>國際通商法律事務所
蔡芸芳<br>TSAI,YUN-FANG	Female<br>女性	Nursing<br>護理	Chang Gung University<br>長庚大學
蕭靜熹<br>CHING-HSI-HSIAO	Female<br>女性	ophthalmology<br>眼科	CGMF (Taipei)<br>台北長庚醫院
邱玟惠<br>WEN-HUI CHIU	Female<br>女性	Law<br>法律	Soochow University<br>東吳大學
葉森洲<br>YEH, SAN-JOU	Male<br>男性	Cardiology<br>心臟內科專科	CGMF (Taipei)<br>台北長庚醫院
陳怡安<br>IAN CHEN	Male<br>男性	Family Medicine<br>家庭醫學專科	National Taiwan University Hospital<br>台大醫院
洪泰和<br>HUNG, TAI-HO	Male<br>男性	Obstetrics and Gynecology<br>婦產科專科	CGMF (Taipei)<br>台北長庚醫院
馮啟文<br>FENG CHI-WEN	Male<br>男性	Ethics<br>宗教	Bread of Life Christian Church in Lin-Kou<br>基督教林口靈糧堂
鄒小蕙<br>Hsiao-Hui Tsou	Female<br>女性	Statistics<br>統計	National Health Research Institutes<br>財團法人國家衛生研究院
郭英調<br>KUO , ING-TIAU	Male<br>男性	Infectious Diseases<br>感染科專科	Chia-Yi Christian Hospital<br>嘉義基督教醫院
廖嘉琳<br>Chialin, Liao	Female<br>女性	LAW<br>法律	Liu & Partners Law Office<br>政勤法律事務所
楊政達<br>YANG, CHENG-TA	Male<br>男性	Pulmonary and Critical Care Medicine<br>胸腔內科專科	CGMF (Taoyuan)<br>桃園長庚醫院

胡幼圃<br>Oliver Yoa Pu Hu	Male<br>男性	Pharmacy<br>藥事	National Defense Medical Center<br>國防醫學院

Chairman: O. T. Hsieh

Tsang-Tang Hsieh,MD
