# Supplementary material for: Frailty Prevention Care Management Program (FPCMP) on Frailty and Health Function in Community-Dwelling Older Adults: A Quasi-Experimental Trial Protocol
Source: Healthcare (Basel). 2023 Dec 17;11(24):3188. doi: 10.3390/healthcare11243188 (PMC10743154; doi:10.3390/healthcare11243188)
Supplement: Supplementary file 1 [file healthcare-11-03188-s001.zip › Supplementary File S3.pdf]

Frailty Prevention Care Management Program.

Intervention group				Control group
12 weeks intervention program	Week	Day1	Day2	Not receive any specific exercise training
	1	Total Resistance Exercises (TRX) program + nutritional assessment	Total Resistance Exercises (TRX) program + nutritional assessment	
	2	Total Resistance Exercises (TRX) program + nutritional assessment	Total Resistance Exercises (TRX) program + nutrition education	
	3	Total Resistance Exercises (TRX) program + nutrition education	Total Resistance Exercises (TRX) program + nutrition education	
	4	Total Resistance Exercises (TRX) program + nutrition education	Total Resistance Exercises (TRX) program + nutrition education	
	5	Total Resistance Exercises (TRX) program + nutrition education	Total Resistance Exercises (TRX) program + nutrition education	
	6	Total Resistance Exercises (TRX) program + nutrition education	Total Resistance Exercises (TRX) program + nutrition education	
	7	Total Resistance Exercises (TRX) program + nutrition education	Total Resistance Exercises (TRX) program + nutrition education	
	8	Total Resistance Exercises (TRX) program + nutrition education	Total Resistance Exercises (TRX) program + nutrition education	
	9	Total Resistance Exercises (TRX) program + nutrition consultation	Total Resistance Exercises (TRX) program + nutrition consultation	
	10	Total Resistance Exercises (TRX) program + nutrition consultation	Total Resistance Exercises (TRX) program + nutrition consultation	
	11	Total Resistance Exercises (TRX) program + nutrition consultation	Total Resistance Exercises (TRX) program + nutrition consultation	
	12	Total Resistance Exercises (TRX) program + nutrition consultation	Total Resistance Exercises (TRX) program + nutrition consultation	
